# Higher fiber intake is associated with lower blood pressure levels in patients with type 1 diabetes

**DOI:** 10.20945/2359-3997000000008

**Published:** 2018-01-01

**Authors:** Mileni Vanti Beretta, Fernanda R. Bernaud, Ciglea Nascimento, Thais Steemburgo, Ticiana C. Rodrigues

**Affiliations:** 1 Hospital de Clínicas de Porto Alegre Divisão de Endocrinologia Porto Alegre RS Brasil Divisão de Endocrinologia, Hospital de Clínicas de Porto Alegre (HCPA), Porto Alegre, RS, Brasil; 2 Universidade Federal do Rio Grande do Sul Universidade Federal do Rio Grande do Sul Departamento de Nutrição Porto Alegre RS Brasil Programa de Pós-graduação em Alimentação, Nutrição e Saúde, Departamento de Nutrição, Universidade Federal do Rio Grande do Sul (UFRGS), Porto Alegre, RS, Brasil; 3 Universidade Federal do Rio Grande do Sul Universidade Federal do Rio Grande do Sul Faculdade de Medicina Departamento de Medicina Interna Porto Alegre RS Brasil Departamento de Medicina Interna, Faculdade de Medicina, Universidade Federal do Rio Grande do Sul (UFRGS), Porto Alegre, RS, Brasil

**Keywords:** Fiber, type 1 diabetes, blood pressure, hypertension

## Abstract

**Objective:**

The present investigation sought to evaluate the potential association between dietary fiber intake and blood pressure (BP) in adult patients with type 1 diabetes (T1D).

**Subjects and methods:**

A cross-sectional study was carried out in 111 outpatients with T1D from Porto Alegre, Brazil. Patients were predominantly male (56%) and white (88%), with a mean age of 40 ± 10 years, diabetes duration of 18 ± 9 years, BMI 24.8 ± 3.85 kg/m^2^, and HbA1c 9.0 ± 2.0%. After clinical and laboratory evaluation, dietary intake was evaluated by 3-day weighed-diet records, whose reliability was confirmed by 24-h urinary nitrogen output. Patients were stratified into two groups according to adequacy of fiber intake in relation to American Diabetes Association (ADA) recommendations: below recommended daily intake (< 14g fiber/1000 kcal) or at/above recommended intake (≥ 14g/1000 kcal).

**Results:**

Patients in the higher fiber intake group exhibited significantly lower systolic (SBP) (115.9 ± 12.2 vs 125.1 ± 25.0 mmHg, p = 0.016) and diastolic blood pressure (DBP) (72.9 ± 9.2 vs 78.5 ± 9.3 mmHg, p = 0.009), higher energy intake (2164.0 ± 626.0 vs 1632.8 ± 502.0 kcal, p < 0.001), and lower BMI (24.4 ± 3.5 vs 26.2 ± 4.8, p = 0.044). Linear regression modelling, adjusted for age, energy intake, sodium intake, and BMI, indicated that higher fiber intake was associated with lower SBP and DBP levels. No significant between-group differences were observed with regard to duration of diabetes, glycemic control, insulin dosage, or presence of hypertension, nephropathy, or retinopathy.

**Conclusion:**

We conclude that fiber consumption meeting or exceeding current ADA recommendations is associated with lower SBP and DBP in patients with T1D.

The American Diabetes Association has specific dietary recommendations for individuals with type 1 diabetes (T1D) (1), which include: preferring whole grains and fiber; preferring fructose naturally current in fruits; restricted intake of saturated fats, trans fats, and cholesterol; and keeping daily sodium intake under 2,300 mg. In addition, regarding fiber in particular, the recommended intake is 14 g total fiber per 1000 kcal of energy intake, or approximately 25 g/day for adult women and 38 g/day for adult men. However, most studies evaluating fiber intake in persons with diabetes have been limited by short observation periods and small sample sizes, and by assessing combinations of fiber-rich diets and foods with low glycaemic index, which may have hindered analysis of the isolated effect of fiber as a determinant of improved glycaemic control in patients with diabetes (2,3).

Some observational studies have demonstrated an inverse relationship between total fiber intake and blood pressure (BP) levels (4,5). Intake of fiber from natural dietary sources, such as whole grains, fruits, and pulses, appears to have a beneficial effect on BP and on serum cholesterol levels in individuals with type 2 diabetes (T2D) (6,7). Furthermore, in patients with T1D and T2D, a high-fiber diet (40 g/day) was associated with significant reductions in fasting and post-prandial blood sugar levels (8). In T1D, dietary fiber plays important roles in lipid profile, glyceamic control, several parameters related to obesity, risk of cardiovascular disease (CVD), endothelial dysfunction, and markers of inflammation (9-12).

Current recommendations for the dietary treatment of hypertension in patients with diabetes, although evidence-based, are largely drawn from studies not conducted in diabetic populations. In fact, the effects of dietary fiber on BP in patients with T1D has been the subject of little research. One recent study of patients with T1D demonstrated a protective effect of fiber, especially soluble fiber, against CVD and all-cause mortality (9).

The role of fiber in the presence of hypertension in patients with T1D has yet to be established definitively. Within this context, the aim of the present study was to evaluate a potential association between dietary fiber intake (in accordance with American Diabetes Association recommendations) and BP levels in adults with T1D.

## SUBJECTS AND METHODS

### Participants

This cross-sectional study was carried out on a cohort of patients with T1D recruited consecutively from the outpatient endocrinology clinic of Hospital de Clínicas de Porto Alegre, a large teaching hospital in Porto Alegre, Brazil. T1D was defined as diabetes with onset before 40 years of age, ketonuria or ketonaemia at the time of diagnosis, and dependence on insulin therapy to sustain life. Patients were selected on the basis of the following criteria: dietary counselling by a dietitian during the 6 months preceding study enrolment; age > 18 years; and duration of diabetes > 5 years. Patients with renal failure, symptomatic heart failure (NYHA class III or IV), acute cardiovascular events in the 6 months preceding study enrolment (stroke, myocardial infarction, or acute pulmonary oedema), or inability to complete weighed dietary records were excluded from the cohort. All ongoing medications were maintained, except for statins.

The recruitment process took place from January 2011 to December 2012, and all participants provided written informed consent for inclusion in the study. The study protocol was approved by the Hospital de Clínicas de Porto Alegre Research Ethics Committee (number 08-470).

### Clinical evaluation

All patients were evaluated by an endocrinologist and interviewed on past medical history, demographic data, medication use, and current lifestyle. Patients were classified by ethnicity as white or non-white. Smoking status was classified dichotomously (as smoker or nonsmoker), as was current alcohol intake (present or absent). The frequency of physical exercise was graded into four levels using a standardised questionnaire, based on activities carried out during a typical day (13); level 1 corresponded to a sedentary lifestyle.

Sitting BP was measured in triplicate, in the left arm, after a 10-minute rest, using a digital sphygmomanometer (Omron^®^ HEM-705 CP). Hypertension was defined as BP readings ≥ 140/90 mmHg on two separate occasions, or current antihypertensive therapy (14). All patients underwent a full physical examination.

The presence of diabetic nephropathy (DN) was classified according to the results of a random spot urine sample or 24-hour timed urinary collection (at least two samples obtained 6 months apart). Patients were considered normoalbuminuric when the urinary albumin excretion (UAE) rate was < 17 mg/L or < 20 μg/min; microalbuminuric when the UAE was 17-174 mg/L or 20-199 mg/min; and macroalbuminuric when the UAE was > 176 mg/L or > 199 mg/min on at least two occasions during a 2-month period (15).

Diabetic retinopathy (DR) was assessed by an ophthalmologist. DR was detected by dilated-pupil direct and indirect ophthalmoscopy, and graded by severity using the Global Diabetic Retinopathy Project Group scale (16) as ‘absence of DR’, ‘mild nonproliferative DR’ (NPDR), ‘moderate NPDR’, ‘severe DR’, or ‘proliferative DR’ (PDR). For purposes of analysis, patients were divided into two groups (absence or presence of DR, regardless of severity).

### Nutritional evaluation

Nutritional evaluation was carried out as described previously (11). Briefly, dietary habits were assessed by means of 3-day weighed food records (two nonconsecutive weekdays and one weekend day), as previously standardised (17,18).

The adequacy of dietary records was evaluated by comparing reported protein intake to protein intake estimated from 24-hour urinary urea nitrogen, collected on the third day of dietary records (17). The ratio of reported protein intake to estimated protein intake was considered acceptable when in the range of 0.79 to 1.26 (18).

The nutrient contents of dietary records were analised in NutriBase 2007 Clinical Nutrition Manager (Cybersoft, Phoenix, AZ, USA), version 7.14 (19). Nutrient intake was expressed as the percentage of total daily energy intake (%) and in grams amounts (g/day or mg/day). Nutrition facts for frequently consumed foods were updated and/or supplemented as necessary with data obtained from local manufacturers of specific industrialized foods.

The total, soluble, and insoluble fiber content was estimated according to data provided in the CRC Handbook of Dietary Fiber in Human Nutrition (20). Dietary sources of fiber were based on the foods described by patients in their 3-day weighed food records. The main sources of dietary fiber consumed by the cohort were pulses (beans, lentils, peas), wholegrain foods (granola, wholemeal bread, wholegrain biscuits/crackers, flaxseed, sesame seeds, sunflower seeds, quinoa, amaranth), rice, barley, potatoes, sweet potatoes, cassava (manioc), eddoes, taro, wheat, and fruits such as apple, banana, clementine, grape, mango, orange, papaya, peach, and pineapple. Vegetables were grouped in two categories on the basis of carbohydrate content (% of grams weight), with group A vegetables containing up to 5% and group B vegetables containing up to 10% carbohydrate (21).

### Laboratory evaluation

HbA1c was measured by high-performance liquid chromatography (Merck-Hitachi 9100; Merck^®^, Darmstadt, Germany) (reference range 4.7-6.0%). Fasting plasma glucose was measured by the glucose-peroxidase enzymatic colorimetric method (Biodiagnostica^®^). Total serum cholesterol and triglycerides were measured by enzymatic colorimetric methods (ADVIA 1800^®^ Autoanalyzer, Germany), and HDL cholesterol by the homogeneous direct method (ADVIA 1800^®^ Autoanalyzer, Germany). High-sensitivity C-reactive protein (hs-CRP) was quantitated by the turbidimetric method (ADVIA 1800^®^ Autoanalyzer, Germany), and fibrinogen was determined by the Clauss clotting method, which measures the rate of fibrinogen conversion to fibrin in a diluted sample under the influence of excess thrombin. Urinary urea was measured by an enzymatic ultraviolet assay (ADVIA 1800^®^ Autoanalyzer, Germany).

### Statistical analyses

Data are presented as mean ± standard deviation (SD), n (%), or median (interquartile range). Baseline characteristics were compared by fiber intake status by means of Student's *t*-test and Mann–Whitney *U* test. Patients were stratified into two groups on the basis of fiber intake in relation to American Diabetes Association recommendations: intake below recommendations (< 14 g/1000 kcal/day) or intake meeting/exceeding recommendations (≥ 14 g/1000 kcal/day). To determine the association between blood pressure and higher fiber intake (≥ 14 g/1000 kcal/day), a linear regression model was constructed with systolic (SBP) and diastolic (DBP) blood pressure values as the dependent variables. The multivariate model was adjusted for age, total calorie intake, BMI and sodium intake. All analyses were carried out in SPSS 18.0 (Chicago, IL, USA).

## RESULTS

Of the 111 patients evaluated, 56% were male and 88% were white. The mean age was 40.0 ± 10.0 years, and mean duration of diabetes was 18.0 ± 9.0 years. Mean BMI was 24.8 ± 3.85 kg/m^2^ and mean HbA1c was 9.0 ± 2.0%. Table 1 describes the clinical and laboratory characteristics of the sample stratified by fiber intake. There were no significant differences between the lower and higher fiber intake groups in terms of age, gender, ethnicity, duration of diabetes, physical activity level, or alcohol consumption. Patients in the high fiber intake group had a lower mean BMI than did those in the low fiber intake group (24.4 ± 3.5 vs 26.2 ± 4.8 kg/m^2^, p = 0.044). There were no between-group differences in waist circumference or presence of hypertension. Regarding BP, patients in the higher fiber intake group (≥ 14 g/1000 kcal/day) had lower SBP (115.9 ± 12.2 vs 125.1 ± 25.0 mmHg, p = 0.016) and DBP (73.0 ± 9.2 vs 78.5 ± 9.3 mmHg, p = 0.009) than patients who consumed less fiber per day than recommended (< 14 g/1000 kcal/day). In addition, patients in the higher fiber intake group used lower doses of insulin. A similar, significant difference was found regarding antihypertensive therapy: fewer patients were on angiotensin-converting enzyme (ACE) inhibitors in the higher fiber intake group than in the lower fiber intake group (11.5% vs 20.8%, p < 0.001). There were no between-group differences in glycaemic control (HbA1c or serum glucose levels), presence of DN or DR, or lipid profile (total cholesterol, HDL cholesterol, LDL cholesterol, and triglycerides).

**Table 1:** Clinical and laboratory profile of patients with type 1 DM, stratified by adequacy of fiber intake (American Diabetes Association) (1)

		Below recommended fiber intake (< 14 g/1000 kcal/day)	At or above recommended fiber intake (≥ 14 g/1000 kcal/day)	P
N		24	87	-
Age (years)		37 ± 13	40 ± 11	0.161†
Sex (male)		29.2%	56.3%	0.180‡
Ethnicity (white)		91.7%	83.9%	0.451‡
Duration of diabetes (years)		19.4 ± 11.0	17.6 ± 8.6	0.394†
Physical activity: level 1* (%)		54.2	46.0	0.811‡
Alcohol intake (%)		54.5	56.2	0.893‡
BMI (kg/m^2^)		26.2 ± 4.8	24.4 ± 3.5	0.044†
Weight (kg)		70.40 ± 12.92	69.02 ± 10.73	0.632†
Height (cm)		162.95 ± 9.15	169.21 ± 9.21	0.004†
Waist circumference (cm)		83.0 ± 8.5	83.3 ± 9.8	0.856†
	Males	78.40 ± 6.22	86.51 ± 9.14	0.059†
	Females	83.30 ± 8.08	79.54 ± 9.44	0.210†
Hypertension (%)		40.9	38.2	0.815‡
SBP (mmHg)		125.1 ± 25.0	115.9 ± 12.2	0.016†
DBP (mmHg)		78.5 ± 9.3	72.9 ± 9.2	0.009†
Under treatment for hypertension		58.3%	32.2%	0.161
	ACE inhibitor	20.8%	11.5%	< 0.001
	ACE inhibitor + diuretic	12.5%	12.6%	0.813
	Diuretic	4.2%	1.1%	0.250
	Calcium channel blocker	4.2%	0.0%	< 0.001
	ACE inhibitor + beta blocker + diuretic	8.3%	2.3%	0.043
Insulin dosage				
	IU/kg body weight	53.88 ± 21.91	47.80 ± 19.34	0.240†
Diabetic nephropathy (%)		0.80 ± 0.26	0.67 ± 0.25	0.061†
Diabetic retinopathy (%)		20.8	12.6	0.312‡
HbA1c (%)		10.7	9.7	0.472‡
Plasma glucose (mg/dL)		90.7 ± 2.5	90.0 ± 1.8	0.079†
Cholesterol, total (mg/dL)		241.33 ± 160.0	193.0 ± 107.0	0.084†
Cholesterol, HDL (mg/dL)		194.2 ± 30	187.3 ± 37	0.408†
Cholesterol, LDL (mg/dL)		60.0 ± 17.0	59.5 ± 16.3	0.884†
Triglycerides (mg/dL)		116.6 ± 31.0	111.5 ± 35.0	0.511†
hs-CRP (mg/L)		98.5 (49-207)	88.1 (34.0-223.0)	0.315†
Fibrinogen (mg/dL)		2.5 (0.1-7.3)	2.1 (0.1-8.4)	0.566†
Urinary sodium excretion (mg/24h)		408.1 (225-672)	364.3 (208-667.0)	0.088†

Data expressed as mean ± SD, median (IQR), or n (%).

† Student's *t* test.

‡ chi-square test.

* Level 1: sedentary.

ACE: angiotensin-converting enzyme; BMI: body mass index; DBP: diastolic blood pressure; HbA1c: glycated haemoglobin; HDL: high-density lipoprotein; hs-CRP: high-sensitivity C-reactive protein; LDL: low-density lipoprotein; SBP: systolic blood pressure.

Daily macronutrient intake values, according to adequacy of fiber intake, are described in Table 2. Patients in the adequate fiber intake group had higher intakes of total energy, carbohydrates, protein, and fat than did patients who consumed less than the recommended adequate intake of fiber. Significant between-group differences were also found in terms of fat fractions (saturated, monounsaturated, and polyunsaturated). Furthermore, patients in the higher fiber intake group had a significantly higher daily intake of sodium (2360.64 ± 919,81 vs 1676.59 ± 688.01 mg/day; p = 0.004). There were no between-group differences in intake of trans fats or cholesterol.

**Table 2:** Daily nutrient intake of patients with type 1 DM, stratified by adequacy of fiber intake (American Diabetes Association) (1)

		Below recommended fiber intake (< 14 g/1000 kcal/day)	At or above recommended fiber intake (≥ 14 g/1000 kcal/day)	P
n		24	87	-
Total energy intake (kcal/day)		1632.8 ± 502.0	2164.0 ± 626	0.001†
Total energy intake (KJ)		6821.76	9045.52	0.001†
Total energy intake (kcal/body weight/day)		24.09 ± 7.3	31.24 ± 8.0	0.001†
Carbohydrate				
	g/day	201.7 ± 71.0	269.6 ± 86.5	0.001†
	% of total energy intake	49.5 ± 7.6	50.2 ± 8.6	0.899†
Protein				
	g/day	74.9 ± 27.1	98.1 ± 33.0	0.002†
	% of total energy intake	18.4 ± 3.5	18.3 ± 3.6	0.742†
Fat, total				
	g/day	57.6 ± 22.8	77.5 ± 34.5	0.009†
	% of total energy intake	31.6 ± 8.3	31.7 ± 9.4	0.952†
Fat, saturated				
	g/day	17.8 ± 9.0	24.2 ± 10.5	0.007†
	% of total energy intake	9.5 ± 2.3	10.0 ± 3.1	0.648†
Fat, monounsaturated		21.0 ± 8.6	27.0 ± 12.6	0.031†
	g/day	21.0 ± 9.8	27.6 ± 12.2	0.021†
	% of total energy intake	9.5 ± 3.1	11.1 ± 3.5	0.627†
Fat, polyunsaturated				
	g/day	12.0 (1.98-23.65)	19.0 (2.7-66.1)	0.026§
	% of total energy intake	7.0 (1.73-12.78)	7.7 (1.8-27.3)	0.519§
Fat, trans (g/day)		0.2 (0.0-2.58)	0.3 (0.0-4.0)	0.770§
Cholesterol, dietary (mg/day)		224.7 ± 171.9	231.3 ± 109.0	0.818†
Sodium (mg/day)		1676.59 ± 688.01	2360.64 ± 919.81	0.004†

Data expressed as mean ± SD or median (IQR).

† *t* test.

§ Mann-Whitney U.

Table 3 describes the daily fiber intake, stratified by type and source, in our sample. As expected, patients in the high fiber intake group (≥ 14g/1000 kcal) consumed more total, soluble, and insoluble fiber than did their counterparts in the below-adequate fiber intake group. Analysis of the main sources of dietary fiber revealed a significant between-group difference in the intake of fiber from pulses. Patients in the high fiber intake group consumed a greater amount of total fiber from pulses than did patients in the below-adequate fiber intake group (7.8 vs 1.6 g/day; p < 0.001). There were no significant between-group differences in terms of total fiber intake from vegetables (groups A+B), tubers, and whole grains.

**Table 3:** Daily fiber intake, types of fiber and their main dietary sources in patients with type 1 DM, stratified by adequacy of fiber intake (American Diabetes Association) (1)

		Below recommended fiber intake (< 14 g/1000 kcal/day)	At or above recommended fiber intake (≥ 14 g/1000 kcal/day)	P
n		24	87	-
Fiber (g/day)				
	Total	10.4 ± 2.0	22.9 ± 7.4	< 0.001†
	Soluble	3.2 ± 1.6	6.5 ± 2.2	< 0.001†
	Insoluble	7.6 ± 2.0	15.8 ± 4.5	< 0.001†
Dietary sources (g/day)				
	Fiber from fruit^a^	1.2 (0.0 – 3.5)	2.7 (0.0-10.2)	0.010§
	Fiber from vegetables (A+B)^b^	1.2 (0.0 – 3.5)	2.8 (0.0-47.2)	0.208§
	Fiber from tubers^c^	0.5 (0.0 – 2.2)	0.5 (0.0-3.5)	0.791§
	Fiber from whole grains^d^	1.5 (0.0 – 6.5)	2.3 (0.0-12.4)	0.292§
	Fiber from pulses^e^	1.6 (0.0 – 5.8)	7.8 (0.0-26.0)	< 0.001§

Data expressed as mean ± SD or median (IQR).

† t test.

§ Mann–Whitney U.

a Fruit: apple, banana, clementine, grape, mango, orange, papaya, peach, pineapple.

b A vegetables: cabbage, chard, cress, cucumber, kale, lettuce, mustard greens, radish, rocket, spinach, turnip.

b B vegetables: aubergine (cooked), beets (cooked), broccoli, carrot (cooked), cauliflower (cooked), chayote (cooked), courgette (cooked), hearts of palm, onion, peppers, runner beans, squash/marrow (cooked).

c Tubers: potatoes, sweet potatoes, cassava (manioc), eddoes, taro.

d Whole grains: granola, wholemeal bread, wholegrain biscuits/crackers, wholemeal flour, oats, flaxseed, quinoa, amaranth, sunflower seeds.

e Pulses: beans, lentils, peas.

Furthermore, we observed inverse correlations between total fiber, soluble fiber, and insoluble fiber intake with BP levels (r = -0.219, p = 0.021; r = -0.221, p = 0.02; and r = -0.215, p = 0.02 respectively).

Linear regression models (Table 4 and Table 5) were constructed to evaluate potential associations between BP levels and higher fiber intake (≥ 14g/1000 kcal). The models were adjusted for age, total energy intake, BMI and sodium intake. Analysis revealed that lower SBP and DBP levels were associated with higher fiber intake (beta -11.11, p = 0.01 and -6.21, p = 0.005 respectively), independent of the adjustments.

**Table 4:** Linear regression analysis: systolic blood pressure and adequate fiber intake (≥ 14g fiber/1000 kcal). Model adjusted for age, total energy intake, BMI, and sodium intake

Variables	β	P
≥ 14g fiber/1000 kcal	-11.11	0.010
Age (years)	0.03	0.810
Total energy intake (kcal/day)	- 0.004	0.200
BMI (kg/m^2^)	0.032	0.930
Sodium intake (mg/day)	0.03	0.031

BMI: body mass index.

**Table 5:** Linear regression analysis: diastolic blood pressure and adequate fiber intake (≥ 14g fiber/1000 kcal). Model adjusted for age, total energy intake, BMI, and sodium intake

Variables	β	P
	-6.21	0.005
≥ 14g fiber/1000 kcal	-0.18	0.040
Age (years)	0.005	0.009
Total energy intake (kcal/day)	0.14	0.550
BMI (kg/m^2^)	-0.001	0.310
Sodium intake (mg/day)	-6.21	0.005

BMI: body mass index.

## DISCUSSION

The present study demonstrated an association between increased dietary fiber intake and lower BP levels in patients with T1D. Patients whose daily fiber intake met or exceeded American Diabetes Association recommendations (≥ 14g/1000 kcal) exhibited lower SBP and DBP levels. These associations remained significant after adjustment for potential confounding factors (age, total energy intake, sodium intake, and BMI). Furthermore, we observed an association between fiber type and BP. Increased intake of soluble and insoluble fiber was negatively associated with BP levels. In this patient group, the mean total fiber intake was 19.8 ± 7.2 g/day (5.8 ± 2.4 g from soluble and 14.0 ± 5.3 g from insoluble fiber). The main sources of dietary fiber associated with significant differences were pulses (beans, lentils, peas) and fruit (banana, apple, papaya, pineapple, clementine, orange, grape, mango, and peach).

The type of dietary fiber appears to play an important role in the risk of cardiovascular disease (CVD) in patients with T1D. Schoenaker and cols. (9) evaluated 2,108 patients with T1D aged 15 to 60 years and found that those who consumed at least 5 g of soluble fiber per day experienced a 16% reduction in CVD risk. In the present study, we evaluated parameters associated with CVD, such as lipid profile, and found no significant association between increased fiber intake (≥ 14g/1000 kcal/day) and serum levels of total cholesterol, HDL cholesterol, or LDL cholesterol.

In this sample of patients with T1D, the higher fiber intake group (≥ 14 g/1000 kcal/day) was also found to have higher energy and macronutrient (carbohydrate, protein, and fat) intake than patients in the lower fiber consumption group (< 14 g/1000 kcal/day). Interestingly, despite this increased energy and macronutrient intake, patients in the higher fiber intake group also had lower BMI, superior metabolic control of diabetes, and inferior use of medications to treat diabetes (insulin) and hypertension (ACE inhibitors). Patients in the higher fiber intake group also consumed more sodium than their counterparts in the lower fiber intake group, although without exceeding the recommended daily intake of sodium for diabetics (22).

Sacks and cols. (23) evaluated the effect of the Dietary Approaches to Stop Hypertension (DASH) diet in hypertensive and normotensive (but nondiabetic) individuals. Those who followed the DASH diet showed significant reductions in BP as compared with those who did not follow the diet. De Paula and cols. (24) analyzed the impact of fiber intake on the BP of 225 patients with T2D. Patients were stratified by BP tertiles, and patients in the subgroup with the highest BP levels (i.e., the top tertile) were found to consume less fruits and vegetables and fewer portions of dairy than recommended in the DASH diet.

A prospective study evaluated the association between quantity and variety of fruits and vegetables in the diet and incidence of diabetes (25), and found that increased intake of these foods was associated with a lower risk of developing any diabetes (21%). Furthermore, increased consumption of fruits alone, vegetables alone, and both fruits and vegetables was associated with significant reductions in the risk of T2D (30%, 22%, and 39%, respectively). In the present study, we observed that fiber from beans/pulses and from fruit was a particularly important dietary component that may be associated with BP levels in patients with T1D.

The mechanisms by which dietary fibers lead to lower BP levels have not yet been elucidated (26). However, is well established in the literature that both soluble and insoluble fibers exert a protective and preventive effect in some pathologies such as metabolic syndrome, CVD and hyperlipidemia (27), in addition, it has an important role as evidenced in satiety and weight loss (28). Dietary fiber intake appears to improve serum lipids levels (29) and this reduction can be associated with improved endothelial vasodilation and consequently with reduced BP levels (27,29). Based on these findings and our observation that the group of patients with higher fiber intake also consumed more energy, including all macronutrients as well as sodium, we suggest that fiber intake may have a favorable effect on vasodilatation and on oxidative stress. Additional studies are needed to elucidate the mechanisms involved in the proposed beneficial effects of fiber intake on BP levels.

A potential limitation of the present study is that BP was only measured during the first study visit and when patients returned to submit their food records. In an ideal setting, 24-hour ambulatory BP monitoring would have been performed. Nevertheless, given the lack of studies demonstrating specific effects of fiber intake on BP level in patients with T1D, our study is highly relevant to clinical practice.

In conclusion, increased total fiber intake is associated with lower SBP and DBP in patients with T1D. Future studies, including randomized clinical trials should confirm the beneficial effects of dietary fiber (of different types and sources) on BP levels in patients with T1D.
